# Supplementary material for: Novel perovskite solar cell with Distributed Bragg Reflector
Source: PLoS One. 2021 Dec 9;16(12):e0259778. doi: 10.1371/journal.pone.0259778 (PMC8659653; doi:10.1371/journal.pone.0259778)
Supplement: S1 Dataset — (PDF) [file pone.0259778.s001.pdf]

T »5, ~6 <601 , PVEP=executed, p 4,  DBR-4, PR Contuinous, op= continous

| | | | | |
|---|---|---|---|---|
| 20.832 | 85.84 | 21.01 | 1.144 | PC=2/1, (4) |
| 21.098 | 86.01 | 21.48 | 1.182 | pc=In correlation w.r.t. geometry |
| 21.558 | 86.48 | 21.96 | 1.201 | |
| 22.075 | 84.57 | 22.03 | 1.209 | |
| 22.625 | 84.42 | 22.67 | 1.226 | |
| 22.849 | 84.97 | 22.82 | 1.264 | |
| 23.01 | 85.32 | 22.95 | 1.284 | |
| 23.14 | 85.87 | 23.42 | 1.325 | |
| 22.99 | 85.74 | 23.01 | 1.301 | |
| 22.87 | 85.55 | 22.98 | 1.296 | |
| 22.71 | 85.42 | 22.84 | 1.287 | |
| 23.14 | 85.96 | 23.63 | 1.329 | |
| 23.16 | 86.04 | 23.95 | 1.401 | |
| 23.19 | 86.07 | 24.01 | 1.404 | |
| 24.09 | 86.18 | 24.38 | 1.409 | |
| 19.54 | 84.76 | 20.58 | 0.998 | |
| 19.89 | 84.94 | 20.73 | 1.054 | |
| 20.43 | 85.13 | 21.01 | 1.046 | |
| 20.74 | 85.26 | 21.56 | 1.124 | |
| 21.01 | 85.49 | 21.85 | 1.168 | |
| 21.16 | 85.86 | 22.24 | 1.234 | |
| 21.13 | 85.82 | 22.2 | 1.229 | |
| 21.04 | 85.21 | 22.15 | 1.22 | |
| 20.98 | 85.1 | 22.04 | 1.213 | |
| 20.96 | 84.56 | 21.92 | 1.205 | |
| 20.84 | 84.01 | 21.76 | 1.196 | |
| 21.19 | 85.94 | 22.56 | 1.239 | |
| 22.03 | 86.06 | 22.72 | 1.302 | |
| 22.09 | 86.15 | 22.99 | 1.316 | |
| 23.12 | 86.23 | 23.03 | 1.321 | |
| 19.243 | 83.84 | 18.06 | 0.847 | |
| 19.254 | 84.01 | 18.48 | 0.878 | |
| 19.301 | 84.08 | 19.03 | 0.894 | |
| 19.396 | 84.05 | 19.56 | 0.901 | |
| 19.586 | 84.24 | 19.68 | 0.91 | |
| 19.748 | 84.54 | 19.94 | 0.931 | |
| 20.028 | 84.6 | 20.01 | 0.949 | |
| 20.242 | 84.5 | 20.06 | 0.968 | |
| 20.398 | 84.92 | 20.45 | 0.984 | |
| 20.649 | 85.48 | 20.67 | 1.002 | |
| 20.442 | 85.42 | 20.54 | 0.992 | |
| 20.842 | 85.58 | 20.98 | 1.044 | |
| 20.982 | 85.89 | 21.03 | 1.109 | |
| 21.253 | 86.01 | 21.14 | 1.152 | |
| 21.424 | 86.06 | 21.28 | 1.168 | |
