# Supplementary material for: Novel perovskite solar cell with Distributed Bragg Reflector
Source: PLoS One. 2021 Dec 9;16(12):e0259778. doi: 10.1371/journal.pone.0259778 (PMC8659653; doi:10.1371/journal.pone.0259778)
Supplement: Attachment — Submitted filename: Response to Reviewers.docx [file pone.0259778.s002.docx]

**Response to Reviewer Comments**

**(Reviewer 1)**

**Reviewer 1:**  The authors have been addressed my questions, the manuscript could be considered to publish after revising the following points:

**Author Reply:** We thank the respected Reviewer for accepting our efforts and suggesting acceptance after minor revisions.

**Reviewer 1:** 1. In Figure 4, DBR1,DBR2,DBR3, DBR4 should be replaced with 1 pair DBR, 2 pairs DBR, 3 pairs DBR,4 pairs DBR.

**Author Reply:** We thank the respected Reviewer for this comment, as these kinds of comments always make the manuscript more worthy and easy for the readers to understand the study more precisely and accurately. As per respected suggestion, Figure 4 has been updated in the revised version of the manuscript.

**Reviewer 1:**  2. In Figure 12a and b, there are no labels for the lines. The different color lines should be labelled clearly.

**Author Reply:** We thank the respected Reviewer for comment. The point is valid, and proper labels for the colored lines have been updated in the revised version of the manuscript.

**Response to Reviewer Comments**

**(Reviewer 2)**

**Reviewer 2:** The manuscript has been improved, but there are same parts need further clarification.

**Author Reply:** We thank the respected Reviewer for observing improvement in the manuscript.

**Reviewer 2:** Equation 7, the symbol for efficiency is not shown properly. I am seeing a question mark inside a box

**Author Reply:** Respected Reviewer, the symbol of efficiency (*ղ*) in Equation 7 is clearly visible in our revised version. There might be some internal error, which is not showing the correct symbol to the Reviewers draft. Once again, we have edited Equation 7, and hope so, it will be visible clearly in the revised version of the manuscript.

**Reviewer 2:** Figure 5, the lines connecting the dots should not be smooth lines. Fig5 (b), the smooth line indicates 2.5 DBR pairs is worse than 2 pairs.

**Author Reply:** We respect the Reviewer's point of view regarding Figure 5 (b). The plot is made in the latest version of the Origin PRO, and the B-Spine option is used for the presented Data. There are no 2.5 pairs used. The number of DBR is as follows 1, 2, 3, and 4. The slight decay between 2 and 3 pairs is the slope that software has produced to give a smooth graphical line.

**Reviewer 2:** Figure 12, legend is need for plots (a) and (b). what does each coloured lines mean?

**Author Reply:** We thank the respected Reviewer for this comment. The point is valid and Figures 12 (a) and (b) have been updated in the revised version of the manuscript. Colored lines represent different cases of the study.
